# Swallowing the Litigation Pill: The Future of Medical Lawsuits in India

**DOI:** 10.7759/cureus.69524

**Published:** 2024-09-16

**Authors:** Satvik N Pai, Madhan Jeyaraman, Naveen Jeyaraman, Sankalp Yadav

**Affiliations:** 1 Orthopaedics, People's Education Society (PES) University Institute of Medical Sciences and Research, Bengaluru, IND; 2 Orthopaedics, South Texas Orthopedic Research Institute (STORI), Laredo, USA; 3 Clinical Research, Virginia Tech India, Dr. MGR Educational and Research Institute, Chennai, IND; 4 Orthopaedics, ACS Medical College and Hospital, Dr. MGR Educational and Research Institute, Chennai, IND; 5 Medicine, Shri Madan Lal Khurana Chest Clinic, New Delhi, IND

**Keywords:** india, law, lawsuits, medical law, medical litigation

## Abstract

As the tide of medical lawsuits rises, doctors in India find themselves navigating increasingly treacherous waters. What drives this surge in litigation? Can we chart a course through these challenging times, or are we destined to face relentless legal battles? In this editorial, we unravel the complexities of this pressing issue and glimpse what lies on the horizon for India's medical community.

## Editorial

Law, lawsuits, litigation, and medical negligence - terms no doctor wants to hear. Yet, terms that we seem to be hearing a lot more of in recent times. The noise around those terms only seems to be increasing. What is happening? Why does it seem like healthcare in India and the law are on a collision course, with doctors feeling like they have been reduced to mute spectators as we move towards that eventuality? Is the quality of healthcare going down? Are the patients being too demanding? Is the law biased? Or is it something else altogether? Here we don the hat of a realist and a soothsayer as we analyze the path we’re on and the road ahead for medical litigation in India.

Where are we right now?

In our interactions with doctors across the country, and across specialties, whenever we discuss the topic of medical litigation in India, many of our senior colleagues in this field have used a word we find fascinating. They tell us healthcare in India is at its crossroads. We have tried to understand what they mean by that and asked them directly as well. As medical men who’ve been in this field for a few decades now, they’ve seen healthcare in India evolve, from a time of limited accessibility to struggles with infrastructure, and now that those hurdles have been crossed to some extent, there seems to be another strong gust of adversity facing doctors in India, that of litigation, and it seems to be gaining speed quite fast. Our colleagues tell us how the medical fraternity is going to face this is going to determine the future of healthcare in the country. We know the destinations it has led to in other countries. As per the Medscape Malpractice Report in 2021, in the United States of America, more than half of the doctors are involved in at least one lawsuit [1]. The number is upwards of 80% among surgeons. That’s the road we’re looking to avoid. Or so we thought. The number of medicolegal cases is increasing at a staggering rate of 110% every year. In 2016, it was estimated that 12% of the cases being fought in consumer courts across the country were medicolegal cases [2]. So to our colleagues who say that we are at a crossroads in terms of medical litigation, we would kindly point out that we are not. We have passed that point a long time ago. We are now well on our way to the ‘highway to hell’.

Can we change courses?

In order for us to be able to steer this ship, we must know what is propelling it forward. There are several factors that are driving this recent surge in medicolegal cases. First, there is just the basic factor of increasing the number of patients. As healthcare reaches more and more individuals, more patients eventually mean the total number of adverse outcomes would increase. While improving the quality of healthcare would decrease the proportion of patients having undesirable outcomes, the nature of the human body and medicine means that not all patients can have the desired outcomes at all times. How many of these less-than-satisfactory outcomes will end up in lawsuits? That proportion is going to increase with the increasing access to legal services. With access to the internet, access to information on one’s legal avenues is at their fingertips, and awareness about consumer rights is on the rise [3]. Like many things in India, cost is always a major factor. With the increasing cost of healthcare, patients’ expectations of outcomes are going to increase proportionally, and sometimes inappropriately [4]. The paradigm shift in the commercialization of the doctor-patient relationship is going to play its role. The laws in the country don’t seem to help much, rather, not help the doctors at least. The legal standing will always favor consumer rights. The inclusion of medical professionals within the Consumer Protection Act puts us in a precarious position, where we are required to practice medicine as a highly specialized and ethical profession but are assessed with yardsticks similar to any other service provider [5]. Lastly, the deficiency in effective communication. Medical education in India has no formal training in communication with patients, and unfortunately, it is something that does not garner the attention it requires later on during practice either. While there may be more factors that may be contributing to the increasing number of lawsuits, we highlighted these to demonstrate that most of the factors, apart from the communication by doctors, are not necessarily within the control of doctors. The lawsuits are only going to keep increasing, despite the efforts of doctors. Again, taking the example of the USA and other developed countries, doctors practicing there are completely aware of the litigious atmosphere and go to great lengths to safeguard themselves from litigation, yet they still face high rates of litigation.

So what do we do?

During a voyage at sea, one is bound to face rough waters at some point, and the only way to survive that is by learning to ride the waves. Every doctor in India must prepare himself mentally and professionally to face at least one litigation at some point in their career. It is highly likely that they will have to, and if they don’t, it’s still better to have been prepared. Having professional indemnity insurance is an absolute must. One must equip oneself with the knowledge of medical aspects of our practice and do the due diligence in ensuring that all statutory requirements are met for our personal licenses and healthcare facilities we may be running. One need not have to practice defensive medicine, but remember that evidence-based medicine is much easier to defend in a court of law than anecdotal experience. I do not agree with the philosophy that every patient must be viewed as a potential litigant, as it would deprive the practice of medicine of its spirit and soul. Every patient must be seen as as an individual with their own rights as a consumer, and having the right on all matters to do with their body, and that right is something we must respect. This burden of litigation need not be borne by each doctor’s shoulder individually. We must be accepting of the reality of litigation and seek to eliminate the stigma or secrecy surrounding it. We must share and discuss medicolegal matters with our colleagues during discussions, conferences, meetings, and even informally. Only when we share experiences and thoughts can we learn from mistakes and collectively work towards a better working environment for all of us. This last point is something I mention in every talk of mine, state in every article, and say to every doctor who’ll lend me their ear. A mountain is not conquered by a lone climber but by the collective footsteps that pave the way.
